# Development of an X-Band Reflectarray Antenna for Satellite Communications

**DOI:** 10.1038/s41598-021-85132-6

**Published:** 2021-03-22

**Authors:** Bing Ma, Fan Lu, Guoping Zhi, Xin Xue, Xiangni Zhao, Chao Ma, Yong Fan, Mei Yang

**Affiliations:** 1 Beijing Institute of Spacecraft System Engineering, Beijing, 100094 China; 2 Beijing Engineering Research Center of EMC & Antenna Test, Beijing, 100094 China

**Keywords:** Magneto-optics, Terahertz optics, Microwave photonics, Micro-optics

## Abstract

An X-band reflectarray antenna using 16 × 12 double square ring elements for satellite communications is proposed in this paper. The feed is a 4 × 3 elements microstrip patch array designed to create edge taper of approximately − 10 dB. A prototype with right-hand circular polarization (RHCP) is manufactured and tested, and the good agreements between simulations and measurements are demonstrated. The good performance is obtained with the aperture efficiency of 40.7% and the 3-dB gain bandwidth of about 10% which is beneficial to nanosatellites.

## Introduction

In the last decade, nanosatellites for low-Earth orbit missions are becoming more and more popular due to their relatively low cost, relatively fast development, and satellite network application capabilities. In order to increase the data transmission speed of nanosatellites, the key requirement is a high gain antenna with a low mass and nearly zero stowed volume. Fortunately the requirement can be met by using a reflectarray antenna, which consists of a feed and flat panels composed of a group of radiation microstrip units. The flat panels can be deployed and folded in a compact way by using hinges. As an effective beam modulation antenna, the reflectarray could transform a spherical wave into a plane wave towards the specific direction by compensating the phase difference between the reflect array units and the feed. There exist several types of reflectarrays such as planar microstrip reflectarrays^1–4^, dielectric resonator reflectarrays^5–7^ and so on. Due to the demand of compact space on nanosatellite, a lighter and thinner circularly polarized reflectarray is more appropriate. The reflectarray antenna can also be utilized in deep space exploration for satellite communications and large aperture SAR antennas.

There mainly exist four kinds of structure to design a reflectarray antenna according to the phase modulation of its elements^16^, such as changing the size of patch units, varying the lengths of slots cut in the patch units, changing the lengths of stubs loaded to patch units and adjusting the angle of rotation of patch units^8–14^. To date, there are mainly two kinds of reflectarray antennas used for satellite communications. The first reflectarray antenna is the Integrated Solar Array & Reflectarray Antenna (ISARA), which incorporates 24 solar cells on the other side of the reflectarray panels and can also provide prime spacecraft power. The ISARA reflectarray design is comprised of three 33.9 cm × 8.26 cm panels and uses square patch elements arranged on a rectangular grid. The measurements indicate that the ISARA reflectarray antenna achieves a > 33.0 dB of gain at 26 GHz, a poor efficiency of 25.2%. The bandwidth is only 0.4% due to using the narrowband square patch elements^15^. The second reflectarray antenna bound for mars is developed in Ref. 17. The measured gain of the antenna is 29.2 dBi at 8.425 GHz, an efficiency of 41.6%. However, the antenna structure suffers from gain bandwidth which is only 0.5%. In order to increase the 1-dB gain bandwidth, Ma uses the double square ring elements with arms of unequal width, and thus a 1-dB gain bandwidth of 25% is reached^16^. However, the reflectarray antenna efficiency is only 24.2%.

This paper presents the recent work on a reflectarray antenna using 16 × 12 double square ring elements for a nanosatellite bus. The feed with left-hand circular polarization (LHCP) is a 4 × 3 elements microstrip patch array designed to create edge taper of approximately − 10 dB. The element with double square rings and a foam layer is used to improve the bandwidth of the reflectarray. Moreover, the broadband microstrip patch array feed is designed to mitigate feed deployment complexity and the whole antenna is designed to fit into commercially available nanosatellite buses. The performance and capabilities of the reflectarray antenna are experimentally demonstrated and discussed in this paper.

### Reflectarray antenna design

**Figure 1:**
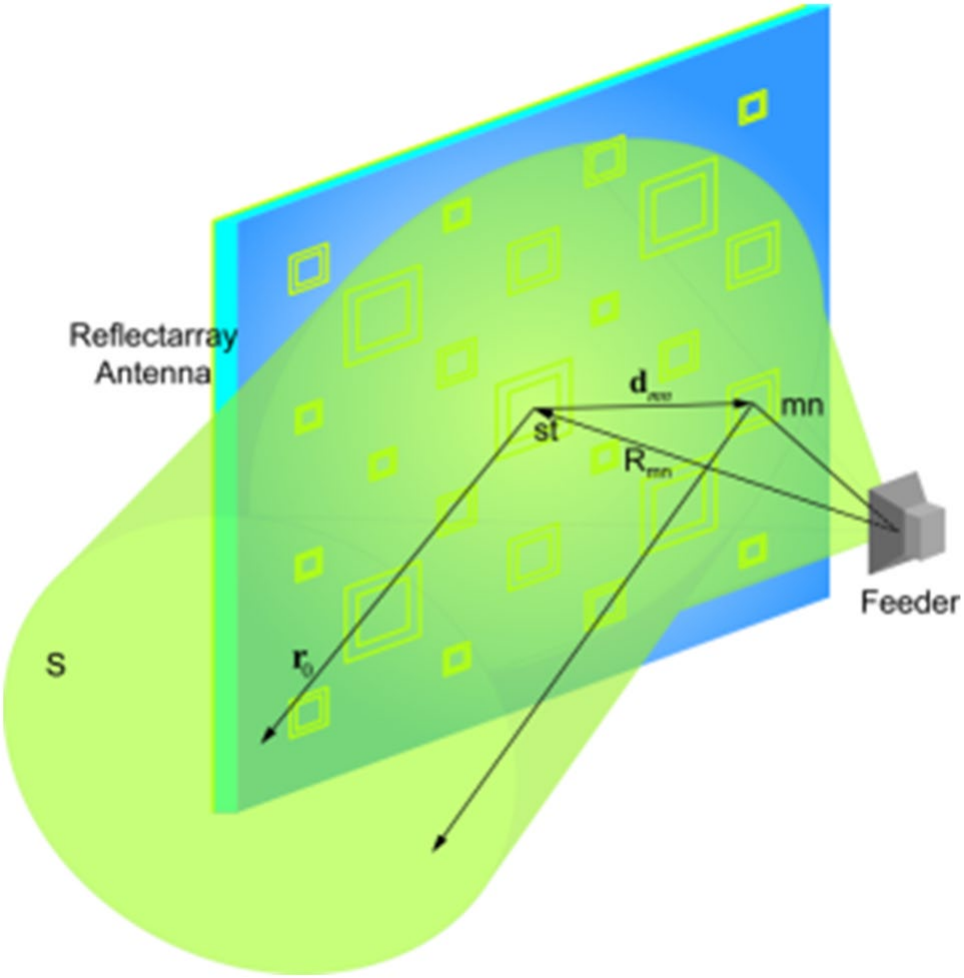
Schematic diagram of the reflectarray antenna.

The basic principle of a reflectarray antenna is illustrated in Fig. 1. The electromagnetic waves are transmitted by the feed and reflected by the plane where each array element is designed to have a proper phase-shift. Then an in-phase radiation in a given direction is formed.

It is supposed that $\varphi_{st}$ is the phase of the wave from the feed to the element *st* and then arriving at the plane *S*, whereas $\phi_{st}$ is the phase delay of the element *st*. Likewise, $\varphi_{mn}$ is the phase of the wave from the feed to the element *mn* and then arriving at the plane *S*, whereas $\phi_{mn}$ is the phase-shift of the element *mn*. Thus, $\phi_{mn}$ is obtained as:

1 $$\phi_{mn} = 2N\pi + k_{0} \left( {R_{mn} - \mathop{d}\limits^{\rightharpoonup} _{mn} \cdot \hat{r}_{0} } \right),N = 0,1,2 \ldots$$

for

2 $$\varphi_{st} + \phi_{st} = \varphi_{mn} + \phi_{mn}$$

where $k_{0}$ is the propagation constant in vacuum, $\hat{r}_{0}$ is the unit vector in the direction of the main reflection beam, $\mathop{d}\limits^{\rightharpoonup} _{mn}$ is the position vector from the center of the reflection plane to the element *mn*, $R_{mn}$ is the absolute value of the vector from the feeder to the unit *mn*. After computing the phase-shift $\phi_{mn}$ from formula (1), the size of the double square ring can be determined due to its phase-shift to accomplish the design of the reflectarray antenna.

**Figure 2:**
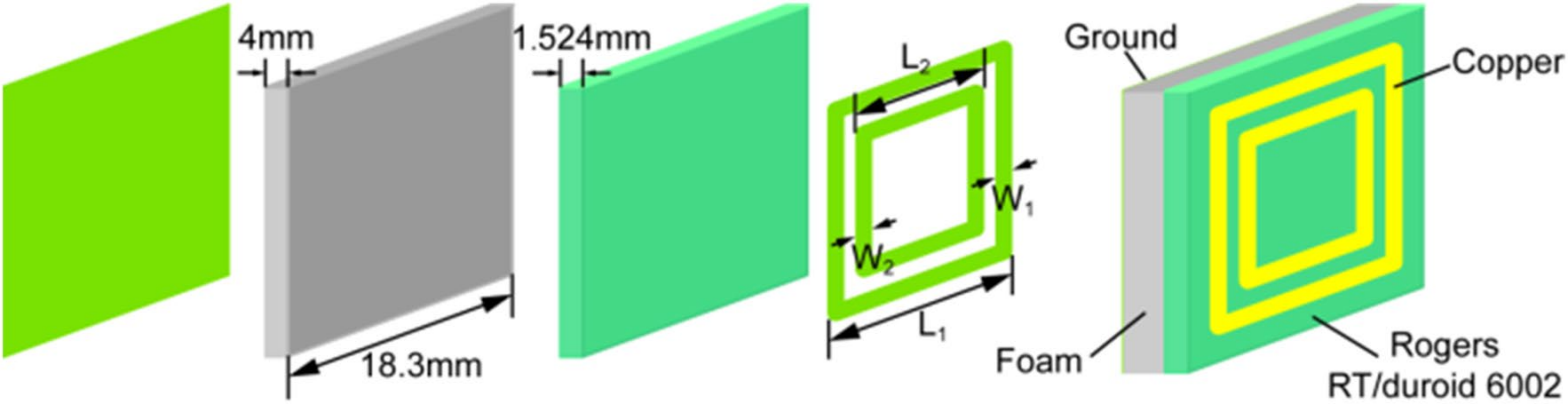
The structural details of the double square ring element.

The element of the reflectarray antenna, which operates at a central frequency of 8.2 GHz, is simple in structure and can be easily manufactured. The patch locates on the top of a commercial substrate Rogers RT/duroid 6002 cube of relative dielectric constant *ε*_r_ = 2.94 and loss tangent tanδ = 0.0012 and consists of double square rings which can increase the phase-shift of the element. When double square rings are used, each of them behaves like a resonant circuit. The phase-shift varies with the length of the ring in a similar way to that of one square ring. Therefore, the phase-shift of double square rings can be increased and the bandwidth can be improved. For the demonstration of this technology, the commercial substrate Rogers RT/duroid 6002 cube is above the low-dielectric-constant foam layer with thickness of 4 mm which can improve the bandwidth of the element. The length and width of the outer square ring are *L*_1_ and *W*_1_ = 0.1 × *L*_1_, while the length and width of the inner square ring are *L*_2_ = 0.65 × *L*_1_ and *W*_2_ = 0.065 × *L*_1_, respectively. The bottom of the element is conducting ground. Figure 2 shows the details of the structure of the double square ring element.

**Figure 3:**
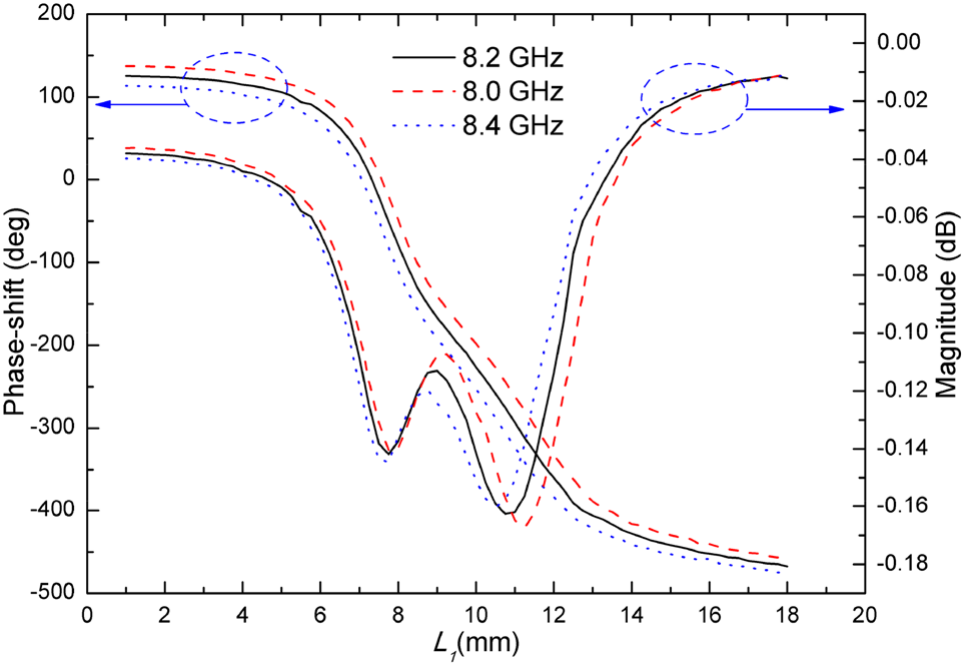
Phase-shift and magnitude curves.

**Figure 4:**
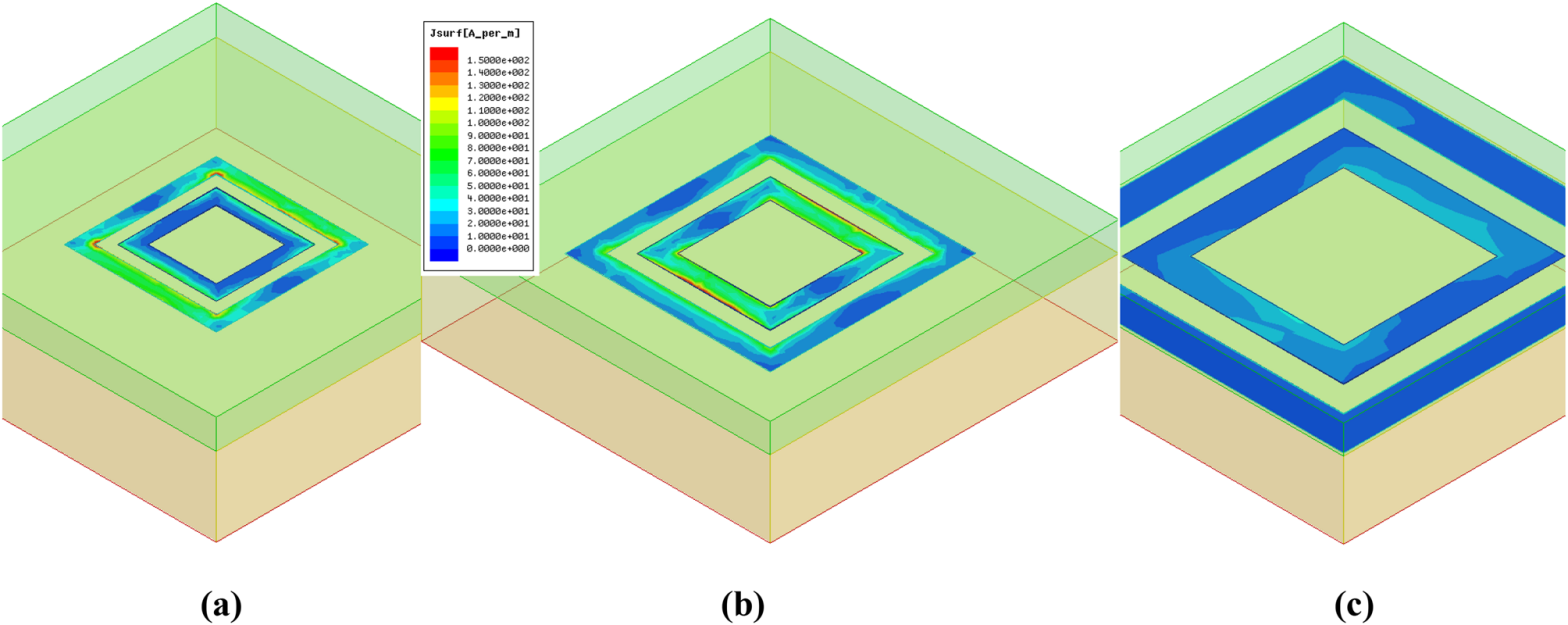
Current distributions on the proposed element at frequency *f* = 8.2 GHz: **(a)** *L*_1_ = 7.75 mm; **(b)** *L*_1_ = 10.75 mm; **(c)** *L*_1_ = 18 mm.

Varying the length of double square rings changes the impedance of double square ring element and therefore the reflection phase-shift is changed. In order to analyze how the reflection phase-shift varies with the size of the double square rings^16^, we suppose that the element is located in an infinite array. Though this is an approximation, it has a tiny effect on the simulation result. In this paper, the simulation software Ansoft HFSS has been used, which affords master–slave boundaries and FloquetPorts to simulate periodic structures. By varying the length of the double square rings it is possible to obtain a phase-shift and a magnitude variation of the reflection field, as shown in Fig. 3. It can be seen that changing the geometric parameter of the square rings results in a phase-shift of more than 570° at frequency *f* = 8.2 GHz, and this variation is nearly linear in the range [110°,-460°], which is beneficial to the precise design of the reflectarray antenna. The performance at frequencies *f* = 8 GHz and *f* = 8.4 GHz is also good which demonstrates a broadband behaviour of the antenna. As clearly shown in Fig. 3, we also notice that there is a change in the reflection magnitude as the length of the outer square ring *L*_1_ is changed. The above method is quoted from the author Ma’s own paper^16^. The magnitude of the reflected wave changes between 0.01 dB and 0.16 dB as a function of *L*_1_ at frequency *f* = 8.2 GHz. When *L*_1_ is 10.75 mm, the maximum magnitude of the reflected wave is 0.16 dB, and the current distribution is shown in Fig. 4(b). The loss is essentially caused by the resonator only. When *L*_1_ is 18 mm, the minimum magnitude of the reflected wave is 0.01 dB, and the current distribution is shown in Fig. 4(c). The current distribution at the second resonance point is shown in Fig. 4(a). Although strong currents are induced on the resonator, the loss remains low due to the good conductivity of the copper and nearly zero loss tangent of the substrate. As the loss is too small it is unnecessary to consider its influence in reflectarray antenna design. The magnitude performance at frequencies *f* = 8 GHz and *f* = 8.4 GHz is similar with that at frequency *f* = 8.2 GHz which also illustrates a broadband behaviour of the antenna.

**Figure 5:**
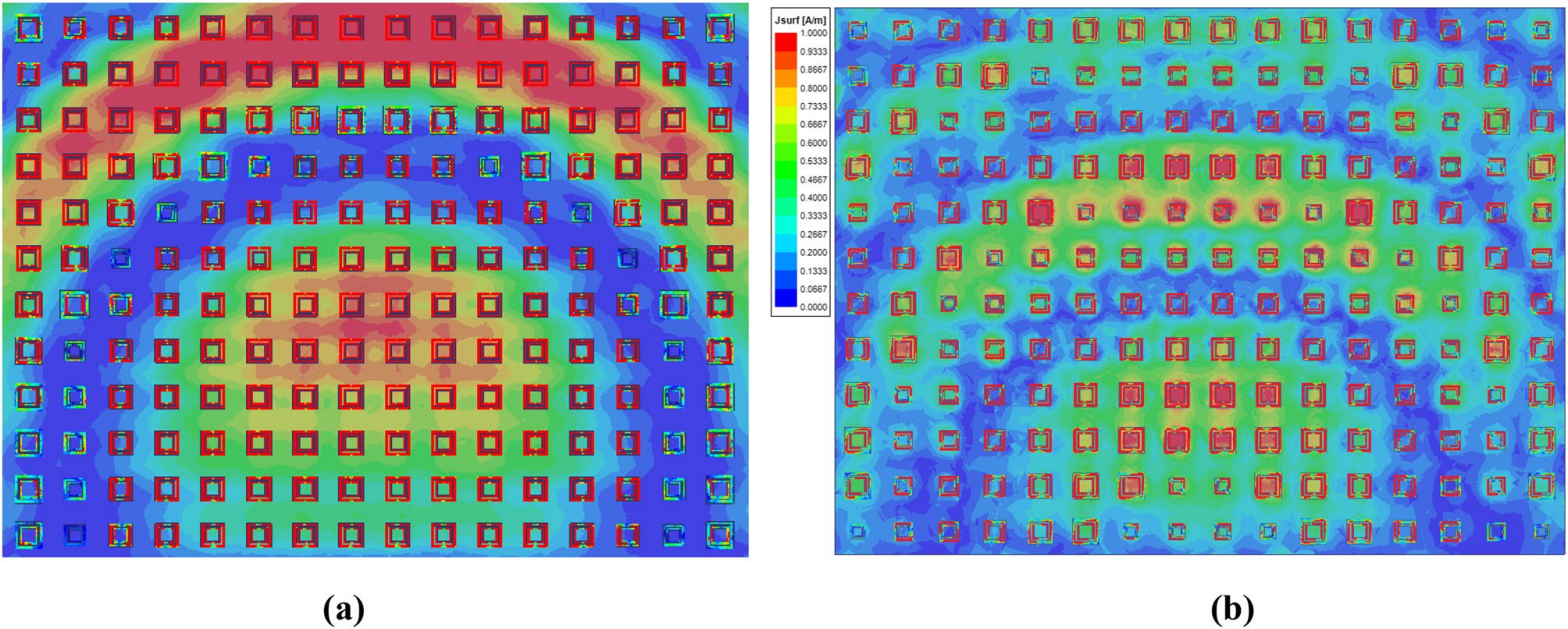
Current distributions on the proposed reflectarray antenna: **(a)** rejected design; **(b)** final design.

**Figure 6:**
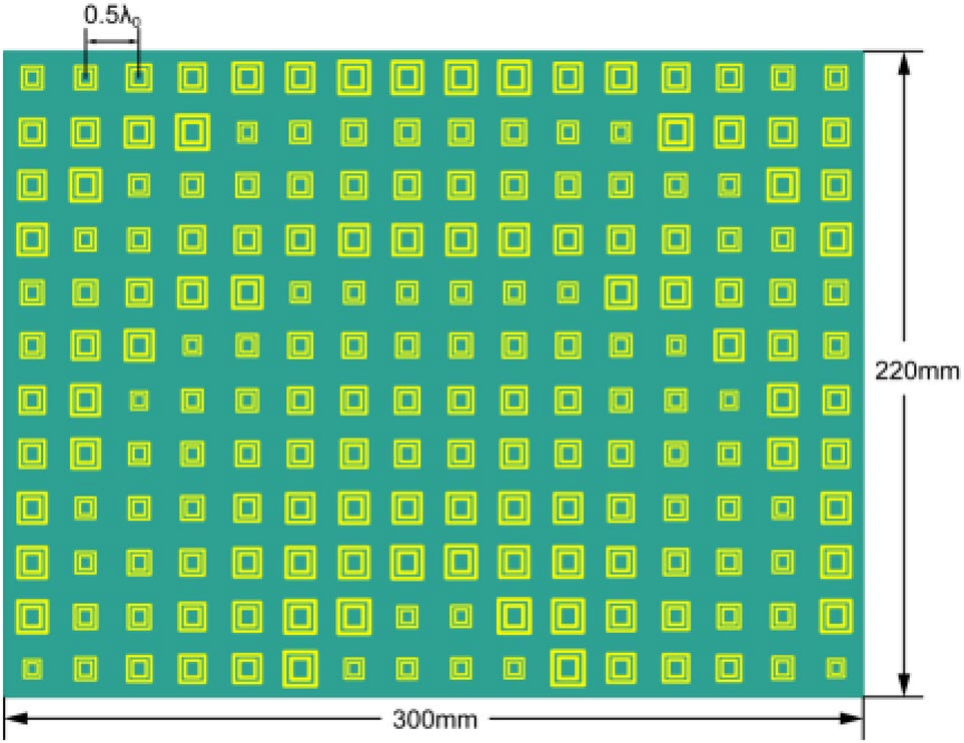
View of the planar reflector.

The dimensions of the double square ring elements are obtained through formula (1) and Fig. 3. Two 16 × 12-element reflectarray antennas are designed at the central frequency 8.2 GHz, as shown in Fig. 5. In Fig. 5(a), the central phase wrap ring is relatively large and the current distribution is extremely nonuniform, while in Fig. 5(b) the current distribution is relatively uniform. By computing the antenna gain and sidelobes, the scheme in Fig. 5(b) is finally adopted. The final view of the planar reflector is shown in Fig. 6. Since the feed is offset, the reflector elements are distributed in circular rings whose center is the center point of the lower edge of the rectangular aperture in Fig. 6. The size of the element is quasi-periodic and varies from 6 to 16 mm. If the element size calculated directly from Fig. 3 is less than 6 mm, it increases sharply and enters the next quasi-periodic cycle.

### Feed antenna design

**Figure 7:**
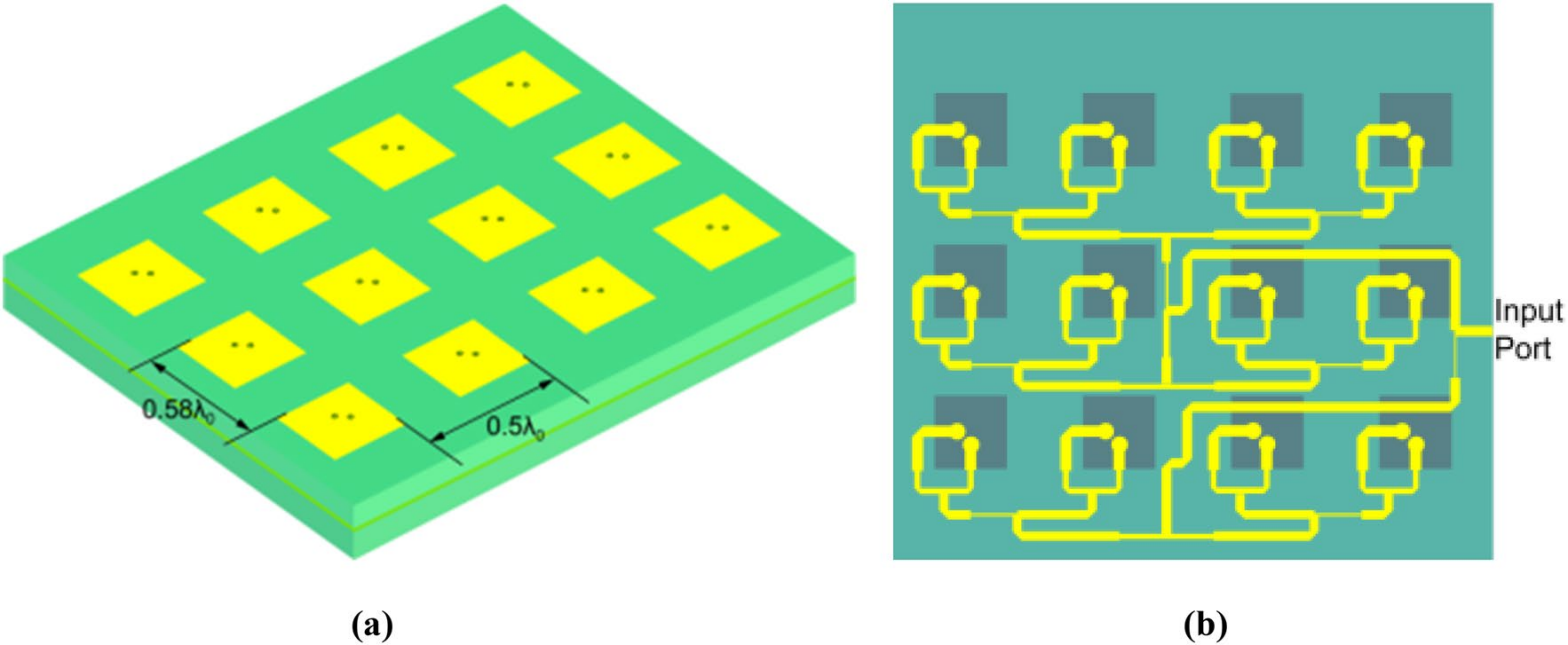
Feed antenna: **(a)** the 45° view and **(b)** the down view.

In order to deploy the feed antenna by hinges in the follow-up practical project, we design the feed as a low profile lightweight antenna. The offset feed is a 4 × 3 elements microstrip patch array designed to create edge taper of approximately − 10 dB, which is shown in Fig. 7. The edge taper angle is set by the reflectarray antenna optics design. A − 10 dB beamwidth of 45.2° in elevation and 68.6° in azimuth is required to minimize spillover and taper loss. Each element of microstrip array antenna is fed by two feeding points, which is helpful to expand the antenna bandwidth. The feed is left-hand circularly polarized because the whole antenna is RHCP. Maintaining good cross-polarization performance over the frequency band is proved to be a significant challenge. The upper substrate with thickness of 1.524 mm and the lower substrate with thickness of 0.508 mm are both Rogers RT/duroid 6002. In order to avoid multipath issues, a -18 dB Taylor distribution is used both in elevation and azimuth through the feeding network.

### Measurement results

**Figure 8:**
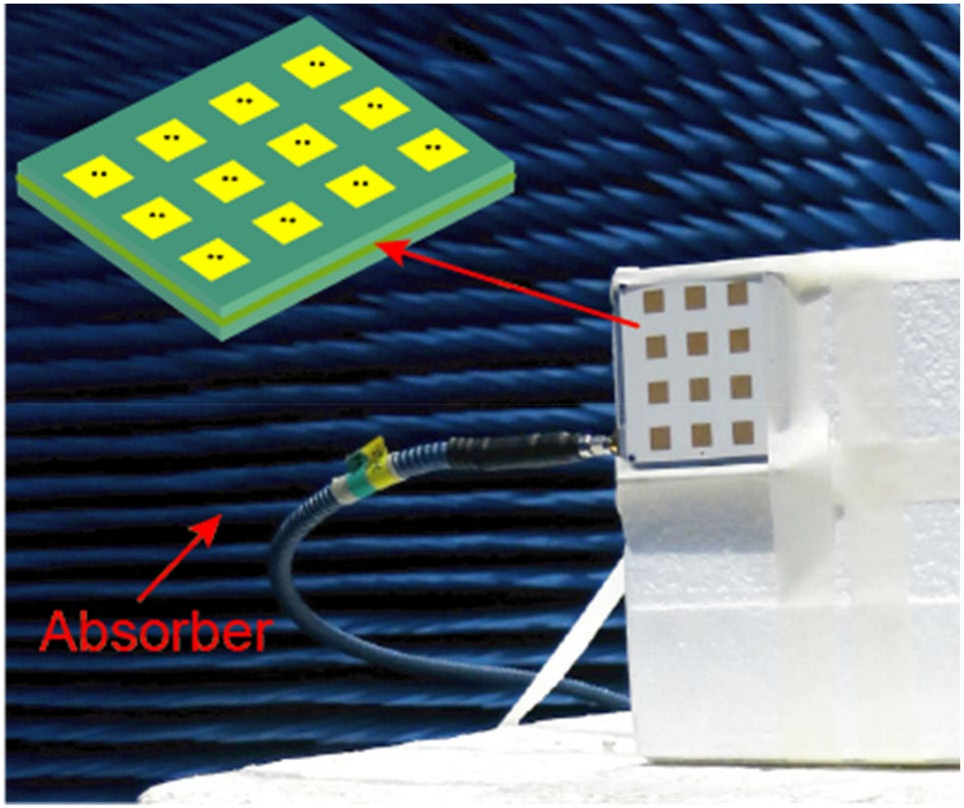
Photograph of the feed array in an anechoic chamber.

**Figure 9:**
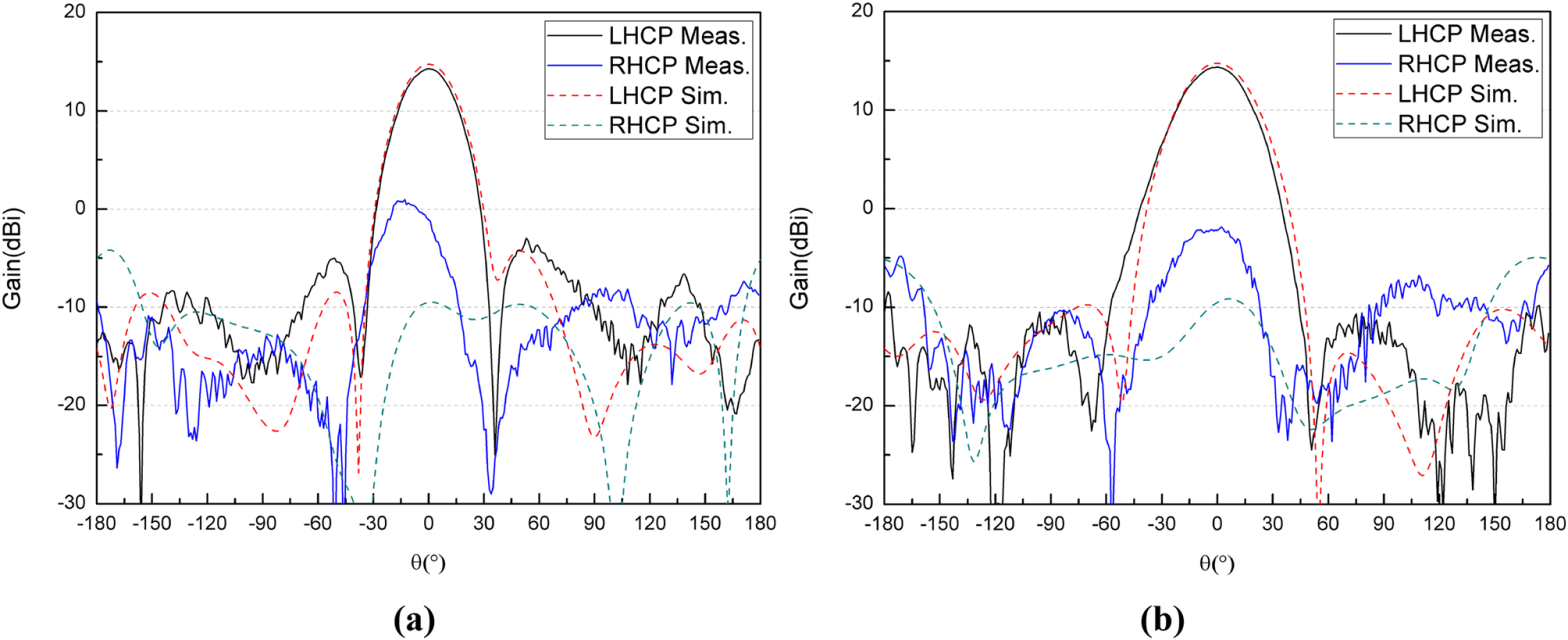
Comparison of measured and simulated gain radiation patterns of the feed: **(a)** the elevation and **(b)** the azimuth.

The microstrip patch array is processed using the conventional photo-etching techniques and measured in an anechoic chamber, as shown in Fig. 8. The simulated and measured results of the feed array are compared as shown in Fig. 9. It could be seen that the simulated and measured co-polar components are in good agreement, and the measured gain is 0.4 dB lower than that of simulation. However, the deviation of cross polarization patterns between simulation and measurement is larger than 9 dB. These discrepancies may be explained by process errors of the photo-etching techniques for minimum precision of 0.05 mm and feeding errors. The measured weight of the feed array is 46 g, and the outer envelope size is 74 × 80 × 7mm^3^.

**Figure 10:**
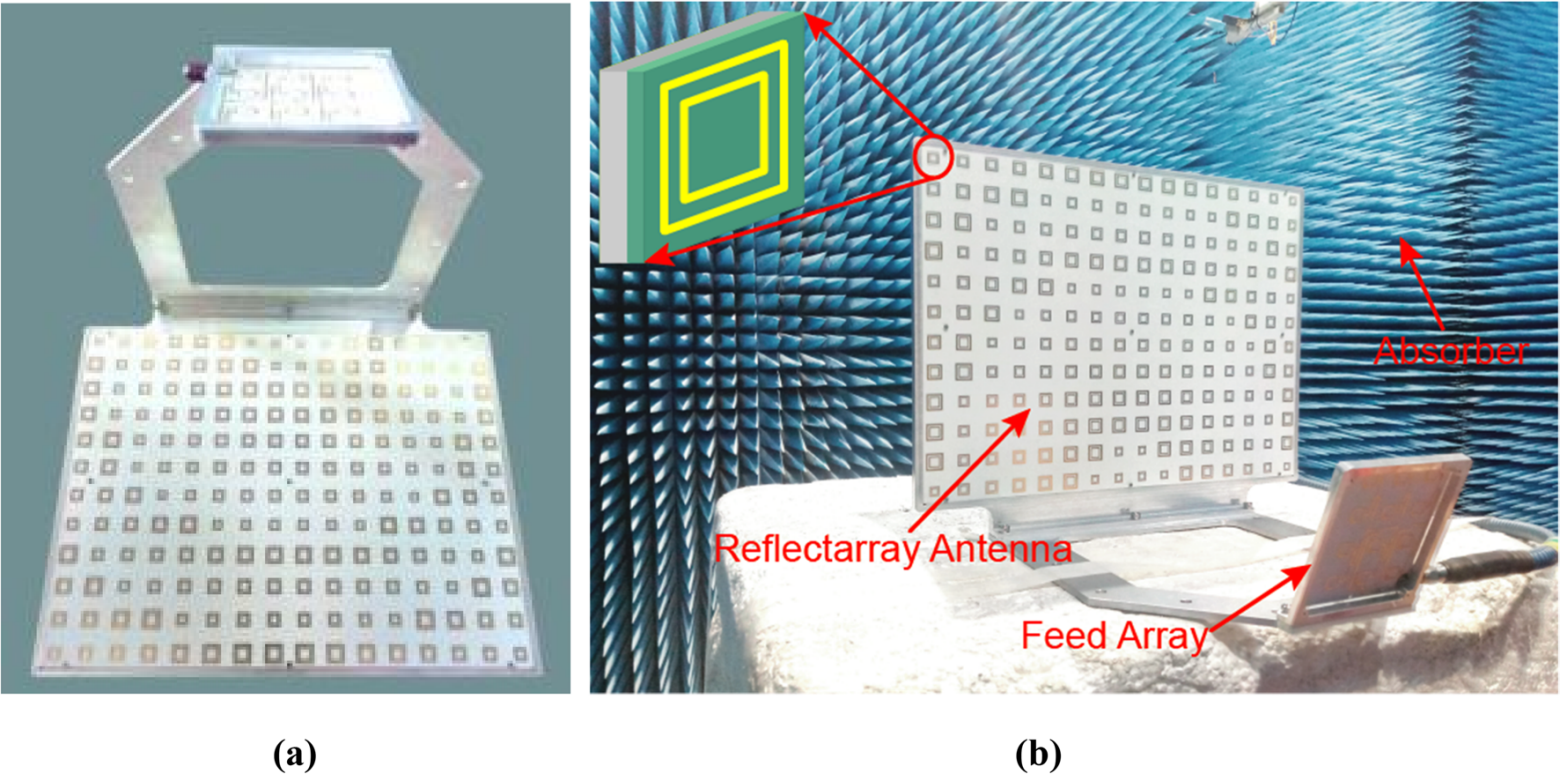
Photograph of the reflectarray antenna: **(a)** the prototype and **(b)** the prototype in an anechoic chamber.

A whole antenna has been manufactured and a photograph of the antenna is shown in Fig. 10 (a). The feed is mounted on a metal plate that is used to support the whole antenna and perform gain radiation pattern tests. The weight of the reflectarray panel assembly is 790 g, and the metal plate is 190 g. The total mass of the whole antenna including the feeder is 1026 g, and the envelope size of the whole antenna is 300 × 226 × 250mm^3^.

**Figure 11:**
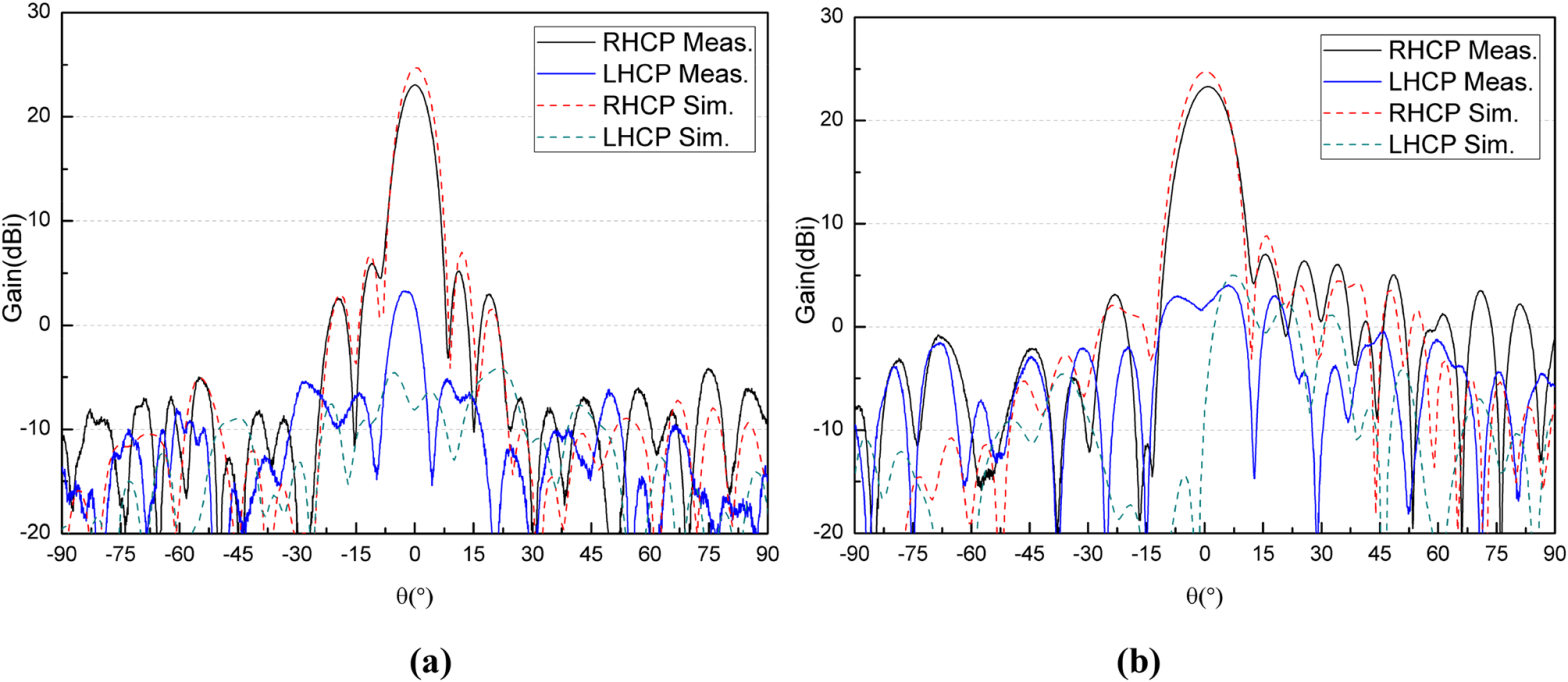
Comparison of measured and simulated gain radiation patterns (8.2 GHz): **(a)** the elevation and **(b)** the azimuth.

**Figure 12:**
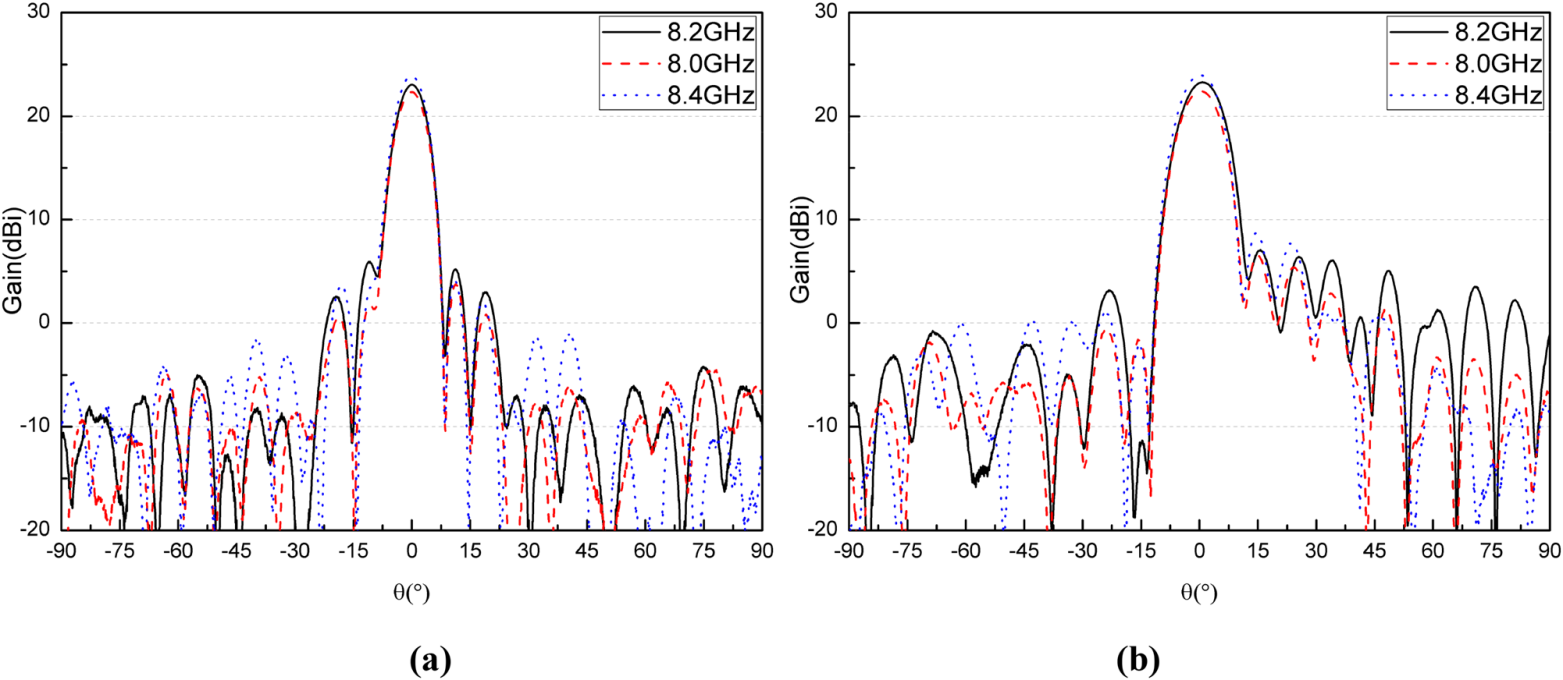
Comparison of measured gain radiation patterns at three frequencies: **(a)** the elevation and **(b)** the azimuth.

**Figure 13:**
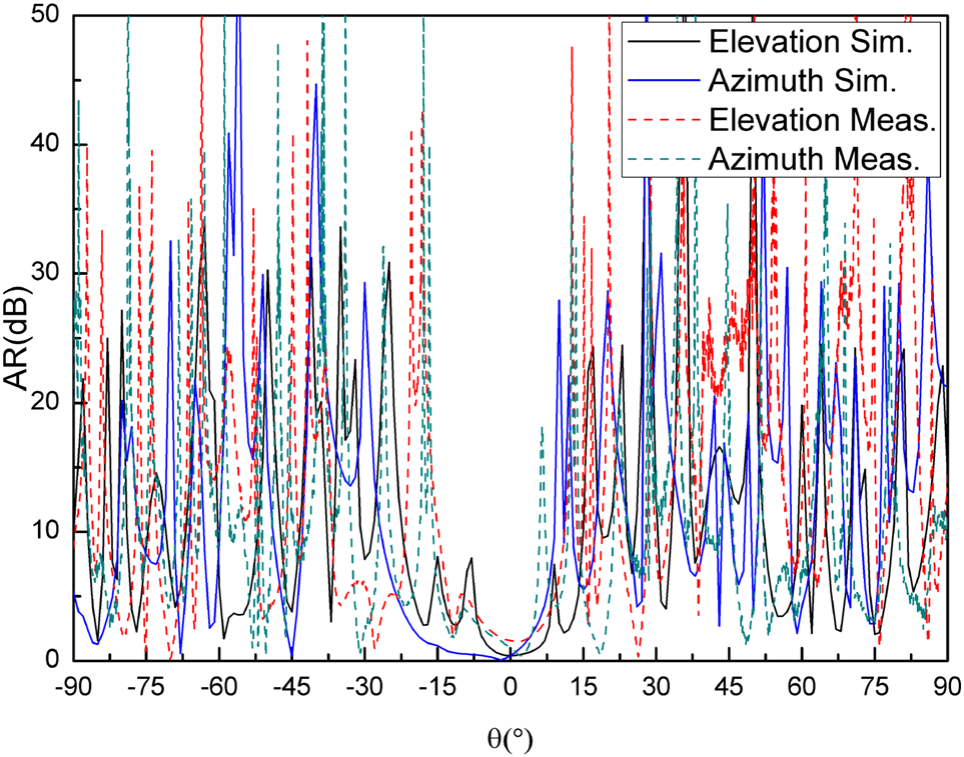
Comparison of measured and simulated axial ratio patterns.

**Figure 14:**
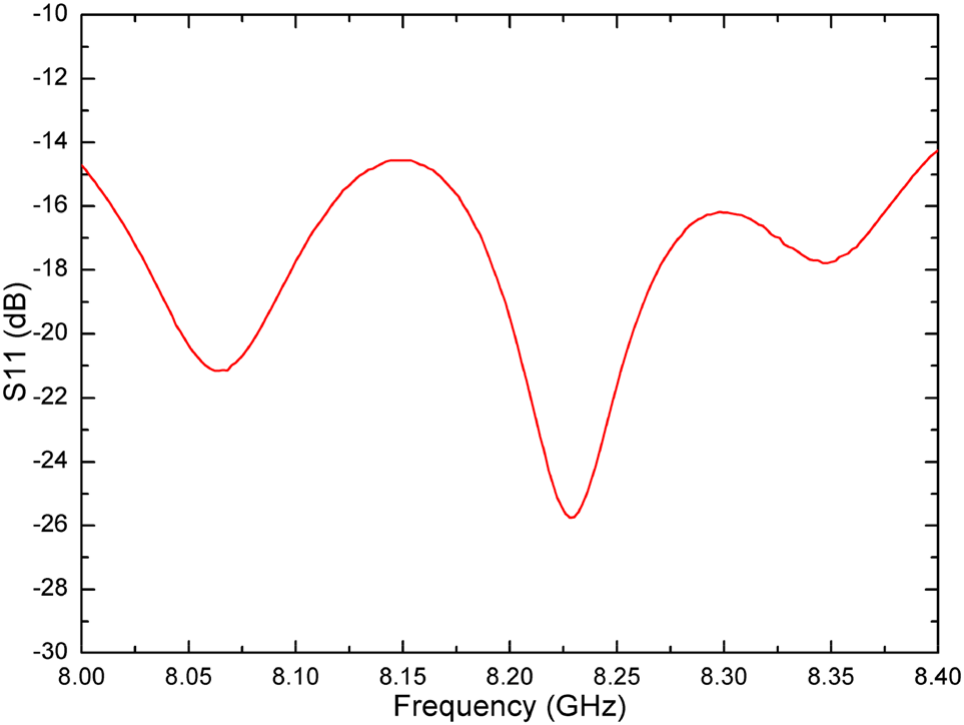
Measured S11 of the reflectarray antenna.

The whole antenna is measured in an anechoic chamber, as shown in Fig. 10(b). The measured and simulated gain radiation patterns are compared as shown in Fig. 11–12. It can be seen that the simulated and measured gain radiation patterns are in good agreement. The measured gain is slightly lower than that of the simulation. These discrepancies may be explained by process errors and dielectric losses. These measurement results indicate that the good fabrication capacity has been achieved. The transmitted efficiency of the reflectarray antenna is 63.9% and the beam transfer efficiency is 63.7%. The transmitted efficiency is low due to the microstrip patch array feed. However, it can be easily deployed in the follow-up practical project. The measured antenna gain gives an aperture efficiency of 40.7% which is mainly caused by using the microstrip patch array feed with a low efficiency, and the 3-dB gain bandwidth is about 10% which is mainly caused by using foam layer and double square metal rings. Figure 13 shows the comparison of measured and simulated axial ratio patterns, which are in good agreement. The measured S11 of the reflectarray antenna is shown in Fig. 14. It can be seen that the achieved S11 is below − 14 dB within the frequency range from 8 GHz to 8.4 GHz.

A summary of the feed and the whole antenna performance characteristics is shown in Table 1. The measured performance shows good agreement with predictions both on feed and the whole antenna. Scattering from the metal plate and the feed can be neglected. A performance comparison between the work of Ref. 17 and this paper is shown in Table 2. It can be seen that the efficiency of the antenna designed in this paper is equivalent to that in Ref. 17. However, the bandwidth of this antenna is much wider than that in Ref. 17. It can be widely used in nanosatellite bus for high-speed data transmission and satellite communication.

**Table 1:** Antenna performance summary.

		Feed		Whole antenna	
		Predict	Meas	Predict	Meas
Frequency (GHz)		8–8.4			
Polarization		LHCP	LHCP	RHCP	RHCP
Gain (dBi)		14.7(8.2 GHz) 13.4–14.7 (8–8.4 GHz)	14.3(8.2 GHz) 13.9–14.3 (8–8.4 GHz)	24.7 (8.2 GHz) 22.3–25.0 (8–8.4 GHz)	23.9 (8.2 GHz) 22.3–23.9 (8–8.4 GHz)
Beam width (°, 8.2 GHz)	AZ	36.2	34.8	7.33	7.21
	EL	28.6	27.5	10.24	10.58
Peak SLL (dB, 8.2 GHz)	AZ	− 24	− 23	− 19	− 18
	EL	− 18	− 18	− 20	− 20
Axial ratio (dB, 8.2 GHz)	AZ	0.8	3	0.4	1
	EL	0.8	2.8	0.4	1.5
Dimension (mm^3^)		74 × 80 × 7		300 × 226 × 250	
Weight (kg)		0.046		1.026

**Table 2:** Performance comparison between Ref. ^17^ and this paper.

	Ref. 17	This paper
Frequency (GHz)	8.4–8.45	8–8.4
Bandwidth	0.5%	**10**%
Efficiency	41.6%	40.7%
Dimension (mm^2^)	597 × 335	300 × 226

## Conclusions

This paper presents an X-band reflectarray antenna using 16 × 12 double square ring elements for a nanosatellite bus, which shows good performance in gain, radiation pattern and frequency aspects. A prototype covering the frequency range of 8–8.4 GHz is manufactured and tested. The performance is in good agreement between the simulations and measurements. The measured gain of the whole reflectarray antenna is 23.9 dBi at 8.2 GHz, an efficiency of 40.7%. The proposed antenna is simple in structure and can be widely used in nanosatellite bus, as it can be stowed in a dead space between the outside of the bus and the sides of the launch canister.
